# Informal Earth Education: Significant Shifts for Environmental Attitude and Knowledge

**DOI:** 10.3389/fpsyg.2022.819899

**Published:** 2022-05-09

**Authors:** Tessa-Marie Baierl, Bruce Johnson, Franz X. Bogner

**Affiliations:** ^1^Centre of Math and Science Education (Z-MNU), Department of Biology Education, University of Bayreuth, Bayreuth, Germany; ^2^Department of Teaching, Learning and Sociocultural Studies, University of Arizona, Tucson, AZ, United States; ^3^Earth Education Research and Evaluation Team, College of Education, University of Arizona, Tucson, AZ, United States

**Keywords:** earth education, informal learning, environmental attitude, environmental knowledge, Campbell paradigm, Earthkeepers

## Abstract

Environmental education aims to affect environmental knowledge and attitude to ultimately induce pro-environmental behavior. Based on 247 upper elementary school students, we tested the impact of an outdoor-based earth education program on environmental knowledge and attitude with a pre-post design. Both outcome measures were Rasch scales. Environmental knowledge is a composite of 27 system, action, and effectiveness knowledge items, and environmental attitude is a composite of 13 evaluative statements and 11 self-reported behaviors about nature preservation. Our analysis revealed gains in environmental knowledge and attitude. The convergence between knowledge and attitude increased significantly from pre- to post-program, and attitude played a significant role in knowledge acquisition.

## Introduction

For the past decades, the world has been facing increasing environmental challenges of threats, including climate change, resource scarcity, waste accumulation, plastic pollution, and deforestation. Several initiatives attempt to promote environmental awareness and a sustainable lifestyle (UNEP, 1978; UNESCO, 2003), including environmental education at an early age. School curricula have been slow to embrace environmental issues, and short-term interventions in the traditional classroom setting seem to have little impact on long-term pro-environmental shifts, so more intense outdoor programs, such as the Earthkeepers earth education program, become increasingly attractive. Earthkeepers is a 3-day residential earth education program with 1-month or more follow-up activities; it is an informal teaching approach that has shown to result in environmental knowledge and attitude shifts (e.g., Manoli et al., 2007; Johnson and Manoli, 2008; Činčera and Johnson, 2013; Baierl et al., 2021). This study goes a step further than those studies on Earthkeepers to not only test knowledge and attitude shifts but whether they converged over the course of the program, with measurements framed by the Campbell paradigm (e.g., Henn et al., 2019; Baierl et al., 2022), which would support attitude’s critical role for knowledge acquisition.

Earthkeepers is an earth education program framed as a “magical learning adventure” developed for upper elementary school students (Van Matre and Johnson, 1988) that originated in the United States and is implemented in different states and around the world (e.g., Činčera and Johnson, 2013; Baierl et al., 2021). It aims to affect environmental attitude and three types of knowledge (i.e., system, action, and effectiveness knowledge; Frick et al., 2004; Roczen et al., 2014) through a 3-day holistic outdoor experience and 1-month or more follow-up activities, with the goal of participants becoming more inclined to pro-environmental engagement. In the program, participants can earn four *keys* to unlock the secrets of becoming an Earthkeeper, the word itself being an acronym for the main components of the program (knowledge, experience, yourself, and sharing). During the initial 3 days at the outdoor center, learners engage in participatory activities to learn about four ecological concepts, namely, energy flow, cycling of materials, interrelationships, and change, and to experience the natural world to earn the K and E keys. Back at home and school, participants work on lessening their impact on and deepening their feelings for the environment to earn the Y key, and they also share their experiences with others to earn the S key and become Earthkeepers. Teachers and parents support the participants in documenting behavior changes and provide evidence that they engaged in lessening their impact and deepening feelings for at least 1 month, with the idea that doing something regularly for that long can form new habits. The program thus resonates with a long tradition of sustainability education, which aims at balancing knowledge, attitudes, and practices to guide students toward sustainability (Salas-Zapata et al., 2018). Several studies of the Earthkeepers program report knowledge gains (e.g., Činčera and Johnson, 2013; Manoli et al., 2014) and positive attitude changes in 4–6 weeks after the residential program (e.g., Manoli et al., 2007, 2014; Baierl et al., 2021). Earthkeepers thus show promising potential to foster long-term pro-environmental shifts.

There is a growing research body on the environmental knowledge-attitude relationship framed by the Campbell paradigm that points at attitude’s role in knowledge acquisition (e.g., Kaiser and Frick, 2002; Frick et al., 2004; Roczen et al., 2014; Henn et al., 2019; Baierl et al., 2022). Within the Campbell paradigm, one can derive an attitude through self-reported behaviors or opinion-based statements by confining to their underlying goal, rendering goals reflections of attitudes (Kaiser and Wilson, 2019). One behavior can be fueled by several goals (Kaiser, 2021): People could refrain from single-use plastic bags because (1) there is a charge on it, (2) they are inclined toward environmental preservation, or (3) in some countries, it might be socially proscribed to use such bags. Scales should therefore ask for a variety of opinions and behaviors to disentangle an underlying attitude (Kaiser, 2021); in this study, i.e., environmental attitude, which reflects a person’s commitment to nature preservation that becomes manifest in protective behaviors or expressions of support for nature preservation. Such behaviors and opinions come with behavioral costs. Costs are the obstacles a person overcomes to carry out behavior, and those are resources, such as time, money, or energy. Reading books and other materials about environmental preservation requires all three of those resources and is, therefore, a behavior that comes with high costs, whereas separating waste from recyclables requires neither money nor a lot of time if done on a regular basis and if the cultural setting provides for it (e.g., government supplying free garbage bins). Costs can thus be imposed by societal constraints or infrastructure (money, time, effort, convenience, or social expectations) and strongly relate to culture, so ecological behavior is not stable but context-dependent, and behaviors can be ordered in terms of costs (Kaiser et al., 2010). We can therefore assume that people agree with increasingly demanding behaviors in a way that is congruent with their attitude. If a person rejects low-cost behaviors, such as reusing shopping bags or turning off the light when it is needed no more, we can conclude a weak environmental attitude. On the contrary, if a person buys products in refillable packages or with eco-labels and contributes to environmental organizations, we can expect a rather strong attitude.

With a Campbellian perspective on attitude that differentiates behaviors in regard to their costs and people’s likelihood of engagement in increasingly costly behaviors, attitude’s role in learning, i.e., knowledge acquisition as the behavior, becomes more apparent. Then, an attitude affects (1) the probability and (2) the intensity of engaging in learning activities, with knowledge as a somewhat measurable outcome (Henn et al., 2019; Baierl et al., 2022). Following this logic, learning activities can lower costs in two ways: First, through the provision of information, students do not need to make an effort and actively seek information; second, through presenting information in an appealing and exciting way (e.g., acknowledging learning theories, such as the cognitive load theory) that reduce costs (Chandler and Sweller, 1991). It requires, e.g., more cognitive resources if a complex environmental issue is presented in a small-print, long text (i.e., concentration is required to disentangle essential information) than when it is sequenced in blocks, reduced to its essentials, and some information is substituted or embellished by visuals like graphs or pictures. In a Campbellian sense, it would require a stronger attitude to engage in such a small-print, long text, whereas a visually engaging text can capture low-attitude students when the educational goal is to impart and acquire information.

However, information is not all the same but can be separated in system, action, and effectiveness knowledge (Frick et al., 2004; Roczen et al., 2014). System knowledge (i.e., procedural knowledge; Schultz, 2002) corresponds with facts or conceptual understanding (Frick et al., 2004), and in this case, can be knowledge about ecological systems or information on human impact on Earth (Díaz-Siefer et al., 2015). Building on system knowledge, action knowledge covers behavioral choices. A person could know about climate change, e.g., the effects of rising CO_2_ and CH_4_ levels in the atmosphere (system knowledge) but lack knowledge about climate-friendly actions, such as using public transportation instead of your car (action knowledge). Effectiveness knowledge is on a higher level and asks for general gains and environmental benefits. It points at broader impacts (Schultz, 2002) through concepts, such as saving energy and reducing carbon emissions on a larger scale like political regulations, so those could be ordered hierarchically regarding their effectiveness (Frick et al., 2004). All those categories form environmental knowledge, and, synergistically, they can help guide toward environmental preservation. The Earthkeepers program incorporates this framework of environmental knowledge, and in this study, we aim to relate it to environmental attitudes.

## Research Questions

Using a shorter, more practicable version of Baierl et al.’s (2022) scale, which is a Rasch-calibrated compilation of expressions of opinions about nature preservation (Bogner and Wiseman, 1999) and self-reported behaviors to preserve the environment (Kaiser et al., 2007), this study investigated the following questions:

(1) How do knowledge and attitude scores change after participation in the earth education program Earthkeepers?
(2) How do knowledge and attitude scores relate over the course of the Earthkeepers program (T1 = before and T2 = 4–6 weeks after the 3-day residential course)?

## Materials and Methods

### Participants and Procedure

Participants consisted of 247 upper elementary school students (age *M* ± *SD*: 9.38 ± 0.51; *n* = 235, 12 students provided no information on age). School classes in the Tucson area in Arizona, United States, visited the earth education center to participate in the Earthkeepers program as part of their education, so participation was not voluntary for students. Gender showed an almost even distribution with 54.7% (*n* = 135) of females and 41.3% (*n* = 102) of males; 10 students provided no information on gender. All participants completed the paper-and-pencil questionnaire 1–2 weeks before (T1) and 4–6 weeks after (T2) the 3-day Earthkeepers program in Arizona, United States. The students experienced nature holistically on-site (i.e., using their senses to tune in on nature, or inquiry-based hands-on explorations) and learned about their natural environment (e.g., system knowledge: how nutrients cycle, action knowledge: the environmental impact of importing fruits, and effectiveness knowledge: hierarchically listing which common electric devices use most fossil fuels). Following the on-site program, educators helped the students put the newly learned knowledge into practice by choosing at least *one behavior* to reduce their energy consumption (e.g., using a bicycle instead of getting a ride by car) and at least *one behavior* to reduce their material use (e.g., re-using paper that has already been used on one site, or taking a shower instead of a bath). The students were encouraged to implement those individual behaviors for at least 1 month after the residential course and to document whether they met their goals in their training manual. Their parents were introduced to the manual and received a four-page explanation on the Earthkeepers and its follow-up tasks. Those tasks referred to the individual behavior changes and nature experiences, so the students were encouraged to regularly spend time outdoors and reflect on their experiences as well as reduce their energy and material use. The manual provided space for the students to document their behavior changes and for recording their thoughts on their nature experiences. Parents then signed whether those things were done, and the teachers talked to the students about the manual. Teachers and parents thus provided evidence that the participants engaged in pro-environmental behavior for at least 1 month after the residential course, and most participants earned the final keys (Y and S) as part of program completion. This helps investigate long-term effects. Since control groups have repeatedly shown no significant changes in knowledge or attitude while Earthkeepers participants did (Johnson and Manoli, 2008; Činčera and Johnson, 2013; Baierl et al., 2021), a control group was not used in this study.

### Measures

We are interested in environmental knowledge, environmental attitude, and their relationship. Therefore, we used an environmental knowledge and attitude scale.

#### Environmental Knowledge

The scale contained 27 items and covered system, action, and effectiveness knowledge (Frick et al., 2004). In total, 13 items involved facts and concepts about our natural environment, such as how energy flows and the materials cycle. Based on this, five action knowledge items asked about behavior options to turn system knowledge into practice: People can, e.g., know about plastic pollution but to protect nature, they further need to know which behaviors reduce their plastic share. In addition, nine effectiveness knowledge items covered the efficiency of conservation behaviors. Studies showed that all three dimensions are helpful for pro-environmental engagement and that all three dimensions work synergistically (e.g., Roczen et al., 2014), so we merged them for our analyses. The participants answered in a multiple-choice format with three to four answer options. For system knowledge, there was one correct answer to gain one point per item. For action and effectiveness knowledge and in line with a partial-credit model, participants could further gain half-points for a good but not the best answer. For the question “Which item is less harmful for the environment to consume?,” participants gained no point for ticking “farm-raised meat,” half a point for “farm-raised veggies,” and one point for “homegrown foods” (see item 11 and all other knowledge items in the Supplementary Table 1).

We conducted a Rasch analysis for all 27 items (Rasch, 1980 or Wilson, 2005; for computational details, see Adams and Khoo, 2015). The person separation reliability by Wright and Masters (1982) estimates the ratio between actual performance (observed behavior) minus the mean square errors of those estimates and the variance of behavior scores; it indicates that our scale was acceptable (*rel* = 0.63). Item difficulties (δ) showed an even distribution from –2.27 to 2.24 with the mean arbitrarily set at zero (*M* = 0.00, *SD* = 1.03). Person parameters ranged from –1.44 to 3.09 (*M* = 0.56, *SD* = 0.80). Negative numbers indicate easier items and lower person estimation scores, while the higher the number, the more challenging an item or the higher the probability of that particular person to give the correct answer is (Bond and Fox, 2007). A person’s ability score of 0 indicates that a person is likely to answer 50% of all questions correctly. For item fit assessment, mean square values (MS) show the correspondence between our data and the Rasch-model prediction, and infit MS should fall between 0.80 ≤ MS ≤ 1.20 (Bond and Fox, 2007); MS thus indicate the discrepancy between the model’s prediction and the observed data. The knowledge items ranged from the value of MS as 0.86 to 1.27, so they were well within or close to the threshold. For an exhaustive list of items and fit indices, refer to Supplementary Table 1.

Environmental attitude was assessed using 13 items from Bogner and Wiseman’s (1999) scale modified for use with children in the United States (Johnson and Manoli, 2008) and 11 items from Kaiser et al.’s (2007) scale. The item compilation covers opinions about nature preservation (i.e., evaluative statements) and self-reports of past preservation-relevant engagement, and is jointly used to measure environmental attitude. It has been suggested to collapse attitudinal instruments within the Campbell paradigm to one dimension, disregarding whether they relied on evaluative statements or self-reported behaviors (e.g., Kaiser et al., 2013; Kaiser and Wilson, 2019), and Baierl et al. (2022) did so with Bogner and Wiseman’s (1999) and Kaiser et al.’s (2007) scales without the need of a predefined fix set of items; both instruments seem to complement each other as Bogner and Wiseman’s (1999) scale contains items that are easier to agree with (people only have to agree with expressions about nature preservation, which requires on average fewer costs) and Kaiser et al.’s (2007) scale contains preservation-based items that are more demanding (people have to actually perform pro-environmental behaviors, which requires on average more costs), so we tested a shorter and slightly modified version for use with children in the United States.

Our attitude measure consisted of 24 items and was calibrated using the dichotomous Rasch model (Rasch, 1980; Wilson, 2005). The participants responded to each item on a five-point frequency scale ranging from strongly disagree to strongly agree (Bogner and Wiseman, 1999) or never to always (Kaiser et al., 2007), and we reverse-coded six negatively formulated items reflecting an environmentally harmful tendency before the analysis (Supplementary Table 2). We dichotomized those polytomous items with strongly disagree, disagree, and not sure/neutral, or never, seldom, and occasionally representing a low-attitude level, while agree and strongly agree, as well as often and always represent a high-attitude level. Collapsing polytomous scales to a dichotomous format is a well-justified approach to prevent excessive measurement error, particularly in attitude research (Matell and Jacoby, 1971; DeCoster et al., 2009). This way, we assessed environmental attitude through personal parameters, and the higher the score, the stronger the probability for environmental preservation-related engagement—a representation of environmental attitude. Scores ranged from –2.35 to 3.73 (*M* = 0.60, *SD* = 1.01). The separation reliability was reasonably accurate (*rel* = 0.73), and item fit statistics showed a fair model fit with MS ranging from 0.9 to 1.16. Item difficulties (δ) ranged from –1.58 to 2.5, and their perceived costs aligned with previous studies, e.g., trying to persuade parents to buy an energy-efficient car as a rather demanding, and taking a shower instead of a bath as a more effortless behavior (e.g., Kaiser et al., 2007; for all items and further fit statistics, Supplementary Table 2).

### Statistical Analysis

Our data were not normally distributed; since tests of the Statistical Program for Social Sciences (SPSS, 24 version) yielded identical results for all (non-) parametric calculations, we report parametric outcomes to be consistent with most studies having used these instruments. The *T*-tests were used to identify program effects, correlations were used for trends, and regression analyses were used for investigating attitude effects on knowledge (gains) and their convergence. We further used a Rasch model (Maximum-likelihood estimates) and dichotomized the five-point frequency scale for attitude (Rasch, 1980; Wilson, 2005). The probabilistic Rasch measurement acknowledges individual engagement (number of items answered positively) and item difficulty (number of people answering to an item positively) and allocates each participant a score (Bond and Fox, 2007). To conduct the Rasch analysis, we used the software ConQuest (Adams and Khoo, 2015).

## Results

### Promotion of Environmental Knowledge and Attitude

**FIGURE 1:**
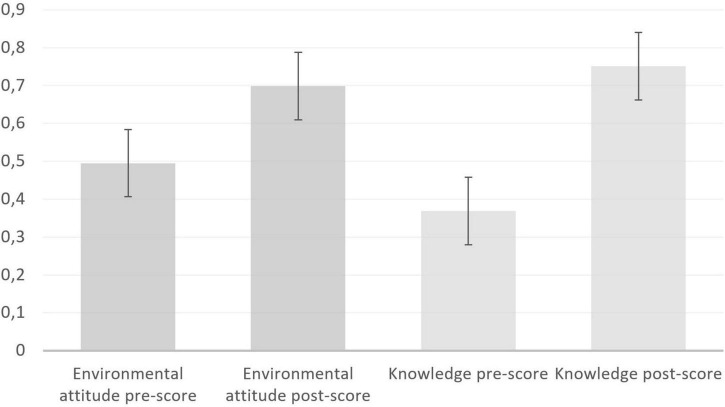
Program effects were statistically significant for environmental attitude and knowledge; error bars indicate 95% confidence intervals. The data were assessed 1 week before and 6–8 weeks after the residential earth education program to determine long-term changes. The *Y*-axis gives the Rasch calibration output, and the higher the score, the more knowledge is prevalent or the stronger the attitude is.

Between T1 and T2, knowledge levels increased, and a *T*-test confirmed statistically significant gains (pre: *M* = 0.368, *SD* = 0.67, 95% CI: 0.28–0.45; post: *M* = 0.751, *SD* = 0.88, 95% CI: 0.65–0.87; *p* < 0.001, *d* = 0.49, *n* = 246). A similar pattern, though the increase was not as large, occurred for environmental attitude (pre: *M* = 0.495, *SD* = 0.86, 95% CI: 0.37–0.59; post: *M* = 0.706, *SD* = 1.12, CI: 0.57–0.85; *p* = 0.001, *d* = 0.22, *n* = 244; Figure 1). There were no considerable gender differences.

### Environmental Attitude as a Lever for Knowledge Acquisition

We correlated knowledge and attitude scores before the program and after the program completion. While both correlations were statistically significant (pre scores: *r* = 0.189, *p* = 0.003, *n* = 246 and post scores: *r* = 0.391, *p* < 0.001, *n* = 244), knowledge and attitude scores correlated significantly stronger after the intervention (*z* = –2.438, *p* = 0.007), which indicates that knowledge and attitude scores converged between T1 and T2. This speaks for a synergistic effect with attitude promoting knowledge acquisition; participants with a stronger pro-environmental mindset seem more likely to learn and to learn more intensely about nature and environmental preservation.

**FIGURE 2:**
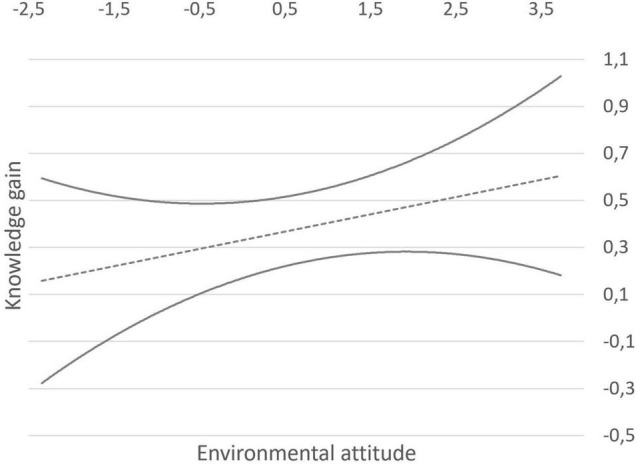
Environmental knowledge gains after the Earthkeepers program regressed on environmental attitude. Their relationship is depicted in the dashed upward trend line. Bold lines show the 95% confidence interval. Both scales are based on a Rasch calibration, so the higher the score, the more knowledge students gained and the stronger the attitude is.

Therefore, we corroborated the dependence of knowledge on attitude levels by conducting a multiple regression analysis. The model showed a high goodness-of-fit (*R*^2^ = 0.239; adjusted *R*^2^ = 0.233; Cohen, 1988). Both knowledge levels before the intervention and attitude levels were statistically significant predictors of post-knowledge scores: *F*(2, 242) = 38.05, *p* < 0.001. While prior knowledge appeared to be the stronger predictor (β = 0.455, *p* < 0.001), attitude levels also showed a statistically significant effect (β = 0.114, *p* = 0.045) on the participants’ post-program knowledge scores. We then regressed knowledge gains on environmental attitude, which revealed a statistically significant effect of attitude on the knowledge participants gained and retained between T1 and T2: β = 0.229; *F*(1, 242) = 13.45, *p* < 0.001; *R*^2^ = 0.049 (Figure 2).

To further test attitude’s role in knowledge acqisition, we tested the role of attitude changes in knowledge gains (i.e., post-program attitude scores minus attitude scores before the program), which turned out to be statistically significant: β = 0.176; *F*(1, 241) = 7.68, *p* = 0.006; *R*^2^ = 0.027. We then tested the effect of prior knowledge scores on attitude changes (β = 0.102), which indicated that attitude increases did not depend on what students knew at the beginning of the program, so attitude gains were not significantly related to prior knowledge levels: *F*(1, 242) = 2.56, *p* = 0.111; *R*^2^ = 0.049. Quite the opposite, i.e., increases in environmental attitudes seemed to promote knowledge gains.

## Discussion

We applied an abbreviated version of Baierl et al.’s (2022) proposed scale that builds on opinion-based expressions to preserve the environment and self-reported preservation-related behaviors to jointly capture environmental attitudes. Our analysis confirmed that environmental attitude’s sound assessment does not depend on a specific set of verbal statements but can be retrieved from various self-reported opinions and behaviors about nature preservation framed by the Campbell paradigm (Kaiser and Wilson, 2019), even with children in a particular environmental setting (i.e., Arizona desert). Based on the scale’s sound calibration, results indicated that it was sensitive to pro-environmental shifts in participants in an earth education program and valuable to knowledge acquisition.

### Effects of the Earth Education Program Earthkeepers

Participants showed pro-environmental shifts in environmental knowledge and attitude 6–8 weeks after the 3-day outdoor earth education program and its one-month follow-up activities. The participants gained and retained knowledge about the environment—a composite of understandings (e.g., how materials cycle and energy flows), knowledge about environmentally friendly behavior options (e.g., how to save water and energy), and general strategies for nature preservation (e.g., how consumer choices affect carbon emissions or waste accumulation). This is in line with previous studies of the Earthkeepers program in different US states and countries (e.g., Činčera and Johnson, 2013; Manoli et al., 2014; Baierl et al., 2021), indicating the outdoor experience and back-in-classroom activities were beneficial in engaging participants in knowledge acquisition. This might be even more important in a society that is faced with growing environmental issues while there is an alienation from nature (Hinds and Sparks, 2008). Pyle (1993, 2003) labeed, and Soga and Gaston (2016) restated and emphasized this trend as the extinction of experience and thus voice the importance of (re-) connecting with nature. This is linked to attitude; pro-environmental shifts can be induced when individuals encounter nature and a sense of connectedness, guided by teachers and parents as role models (Rickinson, 2001; Schultz et al., 2004). Indeed, attitude levels increased over the course of the Earthkeepers program, including not only changes in opinions about nature preservation (assessed *via* evaluative statements) but also became apparent in more frequent environmental engagement (assessed *via* self-reported individual behaviors). Participants changed their perceptions of and engagement in nature preservation. This is underlined by the high rate of participants who earned the Y key, an indicator of frequent pro-environmental engagement based on the newly learned information. Pro-environmental shifts in knowledge and attitude were persistent weeks after program completion, corresponding with other studies about intensive outdoor initiatives (e.g., Bradley et al., 1999; Duerden and Witt, 2010; Fančovičová and Prokop, 2011).

### Knowledge and Attitude Convergence

Environmental knowledge and attitude levels converged over the course of the program, which is before and 4–6 weeks after the on-site course, and further analyses indicated that attitude was a key factor for knowledge acquisition. Though knowledge scores before the program contributed more to knowledge scores after program completion, attitude also turned out to be a significant predictor. It is well known that prior knowledge contributes to gains and retention of topic-related information. Multiple learning theories regard previous knowledge as the basis for effectively gaining new information; e.g., new information is integrated with and linked to pre-existing one, which helps information bridging short to long-term memory (e.g., Chandler and Sweller, 1991). Other theories suggest any sort of knowledge can serve as anchors for new information, even if that knowledge might be, from a scientific view, incorrect. In educational settings, misconceptions can even be integrated into the classroom work to serve as a basis for modification toward scientific more correct information (e.g., White and Gunstone, 1989).

Less apparent is attitude’s role in knowledge acquisition, which is thought to help overcome behavioral costs and thus increase engagement in learning activities, while it further controls the intensity with which participants learn and thus how much knowledge people gain and retain (Henn et al., 2020; Baierl et al., 2022). Our analysis points at both roles of attitude in knowledge acquisition; the stronger the attitude, the more knowledge participants knew before and after the program and the more knowledge participants gained. In a multilevel regression analysis, in addition to prior knowledge, attitude turned out to be a significant predictor of post-program knowledge scores. The stronger the environmentally-conscious attitude, the more participants knew, learned, and retained about the environment. Further robustness tests revealed that increases in attitude scores affected knowledge gains, while prior knowledge scores were statistically insignificant for attitude gains. Although knowledge is a main goal of environmental education, this points to organizing environmental education programs in a way to strengthen attitudes so knowledge follows. This shifts the focus to the question of how environmental attitude can be best promoted, so environmental knowledge follows. The Earthkeepers program seems to be a platform to promote participants’ environmental attitudes, though more research is required as to how attitudes are promoted and how to translate such promotion into the traditional classroom setting.

### Study Limitations

Since we asked for self-reports, social desirability could have affected our findings. However, the original scales have been shown to be resistant to social desirability (e.g., Kaiser, 1998; Oerke and Bogner, 2013). In this study, teachers let participants know that the questionnaires were neither graded nor evaluated. Instead, the questionnaires were filled in anonymously, and the teachers did not look at them but put them in envelopes. Second, though we focused on one outdoor environmental learning center in Arizona, we merged different classes that might have been exposed to different conditions. We tried to compensate by compiling a large dataset to determine general trends. Third, parents and teachers kept track and ultimately signed whether students engaged pro-environmentally and whether they reflected on their nature experiences. This way, as a social contextual factor, parent and teacher involvement might have affected attitude shifts (e.g., Gifford and Nilsson, 2014). Finally, though there have been several studies that found significant program effects in knowledge or attitude in Earthkeepers participants, while control groups showed no such effects, we can only draw assumptions because our study lacked a control group.

## Conclusion

With an environmental attitude measure that builds on preservation-relevant evaluative statements and behavioral self-reports, we were able to document pro-environmental shifts toward more environmentally positive perceptions and a higher frequency of pro-environmental engagement based on the 3-day residential earth education program Earthkeepers with at least 1 month of follow-up activities. The sound assessment of nature preservation, i.e., environmental attitude, does not require a fixed set of items but appears flexible in terms of length, age-groups, and modifications to the specific environment participants encounter, given the composite of opinion- and behavior-based self-reports framed by the Campbell paradigm. Knowledge levels, a composite of facts, understandings, and knowledge about behavior options and general ecological strategies, also increased significantly. Knowledge and attitude levels converged, and in addition to prior knowledge, attitude accounted for post-program knowledge scores, shifting the focus of environmental education programs to strengthening attitudes. This is further supported by a regression analysis showing that the higher the attitude level, the more knowledge participants gained and retained over the course of the Earthkeepers program, which has been demonstrated to be an effective platform for promoting environmental knowledge and attitude.

## Data Availability Statement

The raw data supporting the conclusions of this article can be made available by the authors on request, without undue reservation.

## Ethics Statement

Ethical review and approval was not required for the study on human participants in accordance with the local legislation and institutional requirements. Written informed consent to participate in this study was provided by the participants’ legal guardian/next of kin.

## Author Contributions

T-MB contributed to the conceptualization, methodology, formal analysis, writing the original draft, and visualization. BJ contributed to the resources and writing, reviewing, and editing the manuscript. FB contributed to the writing, reviewing, and editing the manuscript and supervision. All authors have read and agreed to the published version of the manuscript.

## Conflict of Interest

The authors declare that the research was conducted in the absence of any commercial or financial relationships that could be construed as a potential conflict of interest.

## Publisher’s Note

All claims expressed in this article are solely those of the authors and do not necessarily represent those of their affiliated organizations, or those of the publisher, the editors and the reviewers. Any product that may be evaluated in this article, or claim that may be made by its manufacturer, is not guaranteed or endorsed by the publisher.
