# Supplementary material for: Informal Earth Education: Significant Shifts for Environmental Attitude and Knowledge
Source: Front Psychol. 2022 May 9;13:819899. doi: 10.3389/fpsyg.2022.819899 (PMC9126305; doi:10.3389/fpsyg.2022.819899)
Supplement: Supplementary file 1 [file Table_1.docx]

Supplementary Material

# Supplementary Tables

Table 1: Fit statistics of the 27 environmental knowledge items (13 system, 5 action, and 9 effectiveness) ordered in terms of their difficulty

| **Environmental knowledge items** | **Multiple choice answers** | **Item difficulty δ (*SD*)** | **INFIT *MS*** | **OUTFIT *MS*** | **INFIT t** | **OUTFIT t** |
| --- | --- | --- | --- | --- | --- | --- |
| Which of the following is most true? | - Everything is constantly changing - Nothing is changing - Some things change, but some never do | 2.24  (.13) | 1.03 | 1.06 | .5 | .5 |
| Animals use energy that helps their bodies to live and grow. They get that energy directly from: | - Sun and water - Plants and sun - Plants and animals - Water and plants | 1.74  (.11) | 1.03 | 1.12 | .5 | 1.3 |
| Take a look at your pencil. Were the materials that make your pencil ever a part of something else? | - Yes, the materials were once part of something else - No, they were never part of something else | 1.72  (.11) | 1.03 | 1.17 | .5 | 1.8 |
| To lessen our impact on the environment, it makes sense to... | - Drive a new car instead of driving an old car - Eat organic produce - Light a fireplace instead of turning on the heating | 1.67  (.11) | 1.27 | 1.41 | 4.7 | 4.1 |
| Which of the following would there be the greatest number of in a food chain (munchline)? | - Plants - Animals that eat plants - Animals that eat animals - They are all equal | 1.63  (.11) | .91 | .88 | -1.7 | -1.4 |
| People use energy that helps their bodies live and grow. They get that energy directly from: | - Sun and water - Plants and sun - Plants and animals - Water and plants | 1.20  (.10) | .93 | .81 | -.9 | -1.4 |
| The materials that everything is made of: | - Stay in one place forever - Move in a line, never returning to where they started - Move in circles, often returning to where they started | .87  (.10) | .90 | .86 | -3.2 | -2.3 |
| Cotton clothing takes _____ energy to make when compared to synthetic (polyester) clothing. | - The same amount of - More - Less | .83  (.10) | 1.01 | 1.01 | .3 | .2 |
| Importing fruits from South America is bad for the environment because... | - The climate is not good for growing fruit in South America - A lot of packaging material is used - Transporting them to Arizona requires a lot of energy | .73  (.11) | 1.12 | 1.15 | 3.4 | 2.2 |
| Which way is the most environmentally harmful way to go somewhere? | - By car with my mother or father - Using public transportation - Riding a bicyce or walking - Carpooling with friends | .56  (.10) | 1.05 | 1.05 | 1.5 | .8 |
| Why is it better to collect and recycle aluminum cans than to throw it away? | - Because we are running out of materials to make aluminum from - Because discarded aluminum gives off poisons when burned - Because producing new aluminum creates a lot of pollution | .46  (.10) | 1.17 | 1.24 | 5.1 | 3.6 |
| "I can do just one thing without affecting anything else." This statement is: | - True - False - Sometimes true, sometimes false | .09  (.10) | 1.0 | 1.0 | .1 | .0 |
| Where do plants get the energy they need to live and grow? | - Sun - Water - Soil - Sun, water and soil | .04  (.10) | .96 | .93 | -1.0 | -1.1 |
| Which of the following is most true? | - I am made of new materials that have never been part of anything else before - I am made of recycled materials that have been part of other things in the past | -.17  (.10) | 1.12 | 1.13 | 2.9 | 1.7 |
| Imagine a ship has wrecked at sea spilling toxic chemicals into the ocean. Could those chemicals end up inside your body? | - Yes - No - Not sure | -.19  (.10) | .92 | .86 | -2.0 | -1.9 |
| What lunch item uses the most energy and materials? | - Lunchable with ham, cheese, and crackers - Ham, cheese, and crackers in a reusable container - Ham, cheese, and crackers in a plastic bag | -.33  (.10) | 1.22 | 1.34 | 4.8 | 4.0 |
| Which activity uses the most fossil fuel energy? | - Driving a car - Using a cell phone - Lighting a room - Using a computer | -.46  (.11) | .91 | .87 | -1.9 | -1.5 |
| Carpooling is better for the environment than driving by yourself because... | - You get to hang out with friends - It reduces pollution - It is easier to find parking | -.62  (.11) | .90 | .83 | -1.9 | -1.9 |
| Which of the following shows a food chain in proper order? | - Sun 🡪 animals 🡪 plants - Animals 🡪 sun 🡪 plants - Plants 🡪 sun 🡪 animals - Sun 🡪 plants 🡪 animals | -.90  (.12) | .88 | .74 | -1.8 | -2.6 |
| Only _____ can turn sunlight energy into food. | - Animals - Plants - Water - Air | -.95  (.12) | .90 | .79 | -1.5 | -2.0 |
| It is better for the environment to hang your clothes up outside to dry because... | - It uses less energy - You don’t need to spend money on buying a dryer - It makes you go outside to enjoy nature | -.96  (.12) | .86 | .73 | -2.2 | -2.6 |
| Which item is less harmful for the environment to consume? | - Farm-raised meat - Home grown fruits - Farm-raised veggies | -1.23  (.13) | 1.03 | 1.0 | .4 | .0 |
| What is the most important reason to save energy? | - To save money - There is no important reason - To save fossil fuel energy for the next generation - To protect the environment | -1.31  (.13) | .93 | .81 | -.9 | -1.4 |
| Taking a five minute shower uses _____ water than taking a bath. | - The same amount of - More - Less | -1.40  (.14) | .99 | 1.15 | .0 | 1.0 |
| Which yard is the most environmentally friendly in Arizona? | - Desert landscape - Tropical landscape - Grass lawn | -1.49  (.14) | .99 | .88 | -.1 | -.8 |
| If a place is now a desert, | - It could never be an ocean - It may someday be an ocean - It will for sure someday be an ocean | -1.50  (.14) | .91 | .72 | -1.0 | -2.0 |
| Which bag for groceries is the best choice for the environment? | - Plastic bag - Reusable bag - Paper bag | -2.27  (.19) | .95 | .73 | -.2 | -1.2 |
| ***M***  **(*SD*)** |  | **.00**  **(1.22)** | **1.00**  **(.11)** | **.98**  **(.19)** | **.1**  **(2.3)** | **-.1**  **(2.0)** |

:

*Note*: The higher the item difficulty, the more challenging the item was for the participants to answer correctly. Mean square values (*MS*) (unweighted [OUTFIT; 0.60 ≤ *MS* ≤ 1.40] and weighted [INFIT; 0.80 ≤ *MS* ≤ 1.20)] by the item variance) and t values indicate the discrepancy between the model prediction and actual data (Wright & Masters, 1982).

Table 2: Fit statistics of the 27 environmental attitude items ordered in terms of their behavioral costs

|  | **Environmental attitude items** | **Item difficulty δ (*SD*)** | **INFIT *MS*** | **OUTFIT *MS*** | **INFIT t** | **OUTFIT t** |
| --- | --- | --- | --- | --- | --- | --- |
| 1. | I try to persuade my parents to buy an energy-efficient car. | 2.50  (.14) | 1.15 | 1.65 | 1.7 | 3.7 |
| 2. | I ride a bicycle, take the bus, or walk to school. | 1.93  (.12) | 1.13 | 1.46 | 2.1 | 3.6 |
| 3. | For short distances (within 15 minutes) I walk or ride a bike. | 1.48  (.11) | .94 | .96 | -1.2 | -.4 |
| A | *Weeds should be killed because they take up space from plants we need.* | 1.41  (.11) | 1.16 | 1.25 | 3.2 | 2.7 |
| 4. | I have pointed out behavior that is not environmentally friendly to someone else. | 1.16  (.10) | .92 | .92 | -1.9 | -1.0 |
| 5. | I learn about environmental issues through the media (Internet, newspapers, TV). | 1.14  (.10) | 1.09 | 1.18 | 2.0 | 2.2 |
| C | *To feed people, nature must be cleared to grow food.* | .51  (.10) | 1.08 | 1.17 | 2.2 | 2.3 |
| 6. | For making notes, I take paper that has already been used on one side. | .47  (.10) | .90 | .85 | -2.7 | -2.2 |
| 7. | I collect and recycle used paper. | .39  (.10) | .90 | .85 | -3.0 | -2.1 |
| B | *People have the right to change the environment (nature).* | .20  (.10) | 1.14 | 1.25 | 3.8 | 3.1 |
| 8. | I bring empty cans and bottles to a recycling bin. | .08  (.10) | .93 | .89 | -1.7 | -1.4 |
| 9. | I drink water and other beverages in re-usable bottles. | -.15  (.10) | .93 | .83 | -1.7 | -2.0 |
| G | *Because mosquitoes live in swamps, we should drain the swamps and use the land for farming.* | -.17  (.10) | 1.01 | 1.06 | .4 | .7 |
| I | If I ever have extra money, I will give some to help protect nature. | -.43  (.11) | 1.00 | .97 | .1 | -.3 |
| 10. | I reuse shopping bags. | -.46  (.11) | .95 | .87 | -1.1 | -1.3 |
| 11. | If I am the last person to leave a room, I switch off the lights. | -.53  (.11) | .98 | .91 | -.3 | -.8 |
| E | I like to go to places like forests away from cities. | -.70  (.11) | 1.03 | .97 | .6 | -.2 |
| K | I try to tell others that nature is important. | -.86  (.12) | .91 | .83 | -1.5 | -1.4 |
| D | To save energy in the winter, I make sure the heat in my room is not on too high. | -.90  (.12) | 1.04 | 1.13 | .6 | 1.0 |
| M | I would help raise money to protect nature. | -1.12  (.13) | .90 | .73 | -1.4 | -2.0 |
| J | *People are supposed to rule over the rest of nature.* | -1.36  (.13) | .95 | .88 | -.6 | -.7 |
| H | *Building new roads is so important that trees should be cut down.* | -1.49  (.14) | 1.01 | 1.03 | .1 | .3 |
| F | I always turn off the light when I do not need it any more. | -1.52  (.14) | 1.03 | .83 | .3 | -.9 |
| L | I try to save water by taking shorter showers or by turning off water when I brush my teeth. | -1.58  (.14) | .90 | .69 | -1.0 | -1.8 |
|  | ***M* (*SD*)** | **0.00**  **(1.15)** | **1.00 (.09)** | **1.01 (.23)** | **-.1 (1.8)** | **.0**  **(1.9)** |

Note: Items in italics are negatively formulated and were reverse-coded prior to the analysis. Numbers in the left column label items retrieved from Kaiser and Wilson’s (2007) scale, while alphabetical characters show items that originate from Bogner and Wiseman’s (1999) scale. Mean square values (*MS*) (unweighted [OUTFIT; 0.60 ≤ *MS* ≤ 1.40] and weighted [INFIT; 0.80 ≤ *MS* ≤ 1.20)] by the item variance) and t values indicate the discrepancy between the model prediction in relation to the participants’ actual responses (Wright & Masters, 1982).
